# Integrative Bioinformatics: History and Future

**DOI:** 10.1515/jib-2019-2001

**Published:** 2019-09-13

**Authors:** Ming Chen, Ralf Hofestädt, Jan Taubert

**Affiliations:** Zhejiang University, Department of Bioinformatics, Hangzhou, P.R. China; Bielefeld University, Faculty of Technology, Bioinformatics and Medical Informatics Department, Bielefeld, Germany; KWS Saat SE, Einbeck, Germany

## 1 History

The “Human Genome Project” announced the importance of applied computer science for genome analysis. This was the key argument of the German Ministry of Science (BMBF) to support Bioinformatics at the beginning of the 90’s. During the same time the German Society of Computer Science (GI) founded a workgroup (GI FG *Informatik in den Biowissenschaften*) to coordinate national activities. Therefore, the first national conference on Bioinformatics was organized in Bonn 1993. At the same time interdisciplinary activities started across the whole world. For example the first ISMB conference was organized 1993 in Washington. Since 1996 the German annual national conference – the so called German Conference on Bioinformatics (GCB) – became international (the GCB 2019 will be organized in Heidelberg). In parallel the German Society of Computer Science defined the Bioinformatics curriculum and the German Research Foundation (DFG) offered special grants to support faculties building up new studies for Bioinformatics. Furthermore, the German Ministry of Science (BMBF) offered a special grant to support five Bioinformatics Centers in Germany during the same time. Therefore, Bioinformatics was established in Germany and also in many other countries.

From 1995 to 2004 the GI FG *Informatik in den Biowissenschaften* organized different international Dagstuhl seminars, which discussed actual research topics of Bioinformatics. In 1995, the main topic was modeling and simulation based on molecular data and databases. During that time the internet just became common and relevant molecular databases like KEGG, TRANSFAC etc. and information systems like PubMed became available. Having these databases available via the internet, it became important to develop and implement data integration tools. Therefore, computer science developed new data integration methods such as federated databases, data warehouses and text mining. The practical relevance of these techniques was the motivation leading to organization of the Dagstuhl seminar 2004, called seminar of Integrative Bioinformatics. These activities represent the backbone of the online journal Integrative Bioinformatics (JIB), that was founded in 2004. Since 2017 JIB is published by de Gruyter (www.degruyter.com/view/j/jib). Furthermore, the Dagstuhl seminar of Integrative Bioinformatics in combination with the journal Integrative Bioinformatics was also the beginning for the annual conference of Integrative Bioinformatics (IB2019 will take place in Paris: https://symposium.inra.fr/ib2019/). A lot of projects were started and supported by different grants to realize the user specific integration of data. The idea of federated databases was to integrate relevant data coming directly from web based database systems. This method finally failed because of different reasons and data security was/is one key reason. The other research topic during that time was called information fusion. The idea was to integrate user specific data in combination with user specific analysis tools. These research activities induced the data warehouse concept, which allows users to integrate user specific data and analysis tools. The alternative integration concept was and is the specific definition and implementation of workflows. During recent years methods of text mining and data mining got practical relevance. Today such tools, which are able to scan all PubMed abstracts are common and can help to extend the knowledge represented by well annotated database and information systems.

## 2 Future Aspects

We are now increasingly in the big data era. Bioinformatics is facing much more heterogeneous biological data with huge volumes. “Human Genome Projects” like “one million HGP” are going on, leading to more and more individual sequences. It is not only for human, but also for other species, as more and more species have been sequenced. Moreover, it does not simply measure whole tissue samples, but distinctly identify DNAs/RNAs or proteins at a cellular level. Single cell sequencing and single cell proteomics will generate millions of profiling datasets in a short time period. The multi-omics data brings us new challenges to develop appropriate integrative bioinformatics approaches to manipulate, integrate and model complex biological systems at spatial and temporal scales.

Since biological data are subjective and biased, often lacking standardization and reproducibility, and some databases are not well maintained, these resources are becoming more and more degraded. Although there are several bioinformatics methods developed to deal with a certain problem, often only one is widely used and highly cited, which encourages becoming a common/standard method. In many cases, we are not well aware of the original hypothesis of such methods, which may mislead the real problem. How to integrate the multi-omics data with different biological/technical conditions and bias? How to share/deposit data under an acceptable intelligence and ethic policy? Are our traditional data mining and machine learning methods suitable for big data? More powerful tools for multiple scale biological interactome modeling and simulation? How to uncover hidden patterns from such a huge and heterogeneous amount of omics data and allowing the creation of predictive models for real-life applications? Nevertheless, advances in biological technologies and computational methodologies have provided a huge impetus. There are several directions which may lead to break the bottleneck of Integrative Bioinformatics.

1. Integration of multiple biological data towards systems biology. Different omics data is reflecting different aspects of the biological problem. For instance, previously biological networks are regarded as gene regulatory network, protein-protein interaction network and metabolic networks. Now we know that non-coding RNAs, including microRNAs, siRNAs, lncRNAs, ceRNAs and cirRNAs etc. play more important roles in regulations. Therefore, an integrative interactome model (e.g. a virtual cell) of known parts and non-coding RNAs need to be built.
2. Integration of various bioinformatics methods and approaches. Often, to solve a problem, there are many different methods developed by many groups. These methods may perform differently, some good, some bad. However, individual results are often unreliable. In particular cases, the often-used methods may be unreliable or simply ineffective. It is suggested to depend on a variety of results by all methods. With various methods, we are able to integratively develop tailored bioinformatics pipelines to facilitate better understanding of biological problems.
3. To integrate multiple biological data and different methods/approaches, well developed traditional data mining methods such as NN, SVM, HMM are available. However, they are not good enough to deal with high dimensional omics data and big data sets. So far, deep learning methods such as CNN, RNN have been used. Combing with big data, and other approaches, artificial intelligence (AI) has been successfully applied in bioinformatics, especial in the field of biomedical image analysis.
4. Computing infrastructure development. Integrative Bioinformatics in the big data era requires a more advanced IT environment. To facility the related computing and visualization demands, both hardware (e.g. GPU) and software (e.g. Tensorflow) are developing. Supercomputers are used. Cloud services are provided by more and more institutes and big companies.

## 3 Industry Aspects

During the turn of the century the availability of the fully sequenced human genome and other model organisms sparked a boom of bioinformatics companies aiming to address the challenges in medicine, plant and other life sciences using computational methods. Despite initial successes like improving genome annotations or modeling of more complex protein structures, big promises like in-silico drug discovery were not able to be kept and even huge players like Lion Biosciences diminished. Nevertheless, the enthusiasm and learnings of that time led to the establishment of dedicated bioinformatics functions within almost all of life sciences industries. These bioinformatics functions would be placed within the R&D functions of life science companies. As dedicated talent in bioinformatics was rare, biologists, computational scientists or even physicists and others strained in the new area of bioinformatics. The demand of industry for talent influenced the academic world and drove the creation of more and more bioinformatics or related curricular.

As such bioinformatics functions are embedded in the whole R&D ecosystem of a life science company, there are already surrounding data systems in-place concerning relevant bioinformatics data domains. These data systems can range from simple spreadsheets used by the scientists, to Access databases and relational database systems. Understanding the data stored in these systems and adding the contributions of bioinformatics tools and predictions to the R&D ecosystem heavily relies on integrative bioinformatics approaches. Breaking up data silos between functional units within the R&D ecosystem is a prerequisite to drive not only track and traceability of processes but also the discovery of new insights. Technologies like semantic web or linked data provide the base infrastructure of efficient bioinformatics functions. Ontologies either reused from public repositories or customized together with R&D scientists establish a common language, which should also be machine interpretable. FAIR (findable, accessible, interoperable, reusable) principles of data management are more and more being adopted in industry.

Even though hesitant at first, industry is now steadily moving from on-premise data infrastructure to cloud computing. Here the bioinformatics functions are beneath the early adopters of cloud computing as they are commonly exposed to an ever faster changing portfolio of public and proprietary bioinformatics tools and services. As such they rely on the flexibility and power of cloud computing to evaluate new approaches or tools for use in life science industry. Such new approaches may also include artificial intelligence and machine learning techniques. Beside the current hype around these techniques, more and more use cases are being discovered by industry. Here the additional challenge arises to turn a proof of concept into a production ready system to be integrated into the R&D process. This requires not only a sound understanding of the data and algorithms, but also of the end users. Therefore, the classical role of business analysts in industry is supplemented by skills of user centered design and user experience. The outcomes of this interaction are then driving either internal or external software development efforts. To align pre-competitive industry efforts in common tasks of R&D data management, alliances like the Pistoia Alliance (www.pistoiaalliance.org) have been formed. Here, life sciences industry, suppliers, academics and start-ups discuss forthcoming challenges and evaluate common ground.

This special issue presents different views of Integrative Bioinformatics. The paper of Garkov et al. presents an extension of the Vanted system, which represents a well-known integrative system for the analysis of metabolic networks. The paper of Zhang et al. presents relevant results about the integrated networks of ncRNA interactions, providing a comprehensive landscape of ncRNAs regulatory roles. The visualization of metabolic networks and cellular processes is the main focus of the CELLmicrocosmos project, which is the topic of the paper of Björn Sommer. The application paper of Alban Shoshi and Marcel Friedrichs is based on such methods and shows how this kind of data integration can help to solve medical questions. The paper of Jens Allmer discusses the future of Bioinformatics based on the internet of science. Overall education is important and the paper of Bukas et al. shows how interdisciplinary lectures should be created in the future.

